# Multishell Diffusion MRI Reflects Improved Physical Fitness Induced by Dance Intervention

**DOI:** 10.1155/2020/8836925

**Published:** 2020-11-05

**Authors:** Alzbeta Sejnoha Minsterova, Patricia Klobusiakova, Sylvie Kropacova, Lubomira Novakova, Lubos Brabenec, Zuzana Balazova, Roman Grmela, Alena Skotakova, Lenka Svobodova, Irena Rektorova

**Affiliations:** ^1^Applied Neuroscience Research Group, Central European Institute of Technology, Masaryk University, Kamenice 5, 625 00 Brno, Czech Republic; ^2^Faculty of Medicine, Masaryk University, Kamenice 5, 625 00 Brno, Czech Republic; ^3^Psychology Department, Faculty of Arts, Masaryk University, Arne Nováka 1, 602 00 Brno, Czech Republic; ^4^Department of Health Promotion, Faculty of Sports Studies, Masaryk University, Kamenice 5, 625 00 Brno, Czech Republic; ^5^Department of Gymnastics and Combatives, Faculty of Sports Studies, Masaryk University, Kamenice 5, 625 00 Brno, Czech Republic; ^6^First Department of Neurology, Faculty of Medicine, Masaryk University and St. Anne's University Hospital, Pekařská 664/53, 656 91 Brno, Czech Republic

## Abstract

Using multishell diffusion MRI and both tract-based spatial statistics (TBSS) and probabilistic tracking of specific tracts of interest, we evaluated the neural underpinnings of the impact of a six-month dance intervention (DI) on physical fitness and cognitive outcomes in nondemented seniors. The final cohort had 76 nondemented seniors, randomized into DI and control (life as usual) groups. Significant effects were observed between the DI and control groups in physical fitness measures and in attention. We detected associations between improved physical fitness and changes in diffusion tensor imagining (DTI) measures in the whole white matter (WM) skeleton and in the corticospinal tract and the superior longitudinal fascicle despite the fact that no significant differences in changes to the WM microstructure were found between the two groups.

## 1. Introduction

Dance intervention (DI), regardless of the type of dance, was shown to have a positive impact on the overall physical health of older adults [1]. Dancing is an activity that comprises a wide range of skills, including motor learning, action observation, sensorimotor coordination, and synchronization with a group; it engages physical endurance, balance control, motor learning, and cognitive functions [1, 2]. Keogh et al. [3] in their review concluded that dancing can significantly improve the aerobic power, lower body muscle endurance, strength, flexibility, balance, and agility of older adults. Another more recent review [4] proved the beneficial effect of dance on physical fitness (mostly reported as balance and motor skills in general) in patients with different kinds of pathology.

The influence of the different types of dance activity on cognition in healthy seniors has been tested behaviorally [5, 6]. Coubard et al. [5] reported that contemporary dance training (one lesson/week, 5.7 months) leads to improvement in switching attention in older adults. Kattenstroth et al. [6] reported positive effects of regular dancing (one lesson/week, six months) on attention in particular. Neither of these studies examined any possible neuroimaging correlates of the positive effect of DI.

Diffusion tensor imaging (DTI) is the most widely used model of diffusion MRI, which allows the evaluation of changes in the brain microstructure, especially in the white matter (WM) [7]. Voss et al. [8] used conventional single-shell DTI and examined the effect of aerobic fitness training (three lessons/week, one year). The authors observed a relationship between increased aerobic fitness and increased fractional anisotropy (FA) in prefrontal, parietal, and temporal areas. On the other hand, they found no difference in cognitive performance between groups. The only study evaluating the effect of the DI (three lessons/week, six months) in healthy seniors with conventional DTI was performed by Burzynska et al. [9]. The authors used tract-based spatial statistics (TBSS) and selected regions of interest on the TBSS skeleton using the DTI WM atlas. They found that FA in the fornix increased in the DI group as compared to control groups. However, the change in fornix integrity did not correlate with the change in cognitive outcomes. In fact, there were no significant cognitive changes during DI surpassing changes in the control groups.

In our previous paper [10], we demonstrated subtle effects of an optimized, structured six-month dance intervention on executive functions in aged people without dementia, particularly in the Five-Point Test (FPT) which evaluates attention and executive functions [11]. In the current DTI substudy, we aimed to explore the neural correlates of the DI-induced changes in physical fitness and cognition using a multishell diffusion MRI protocol. The use of multiple shells improves the modeling of crossing fibers within each voxel [12]. Moreover, by quantifying the non-Gaussian diffusion, the multishell DTI provides more precise information about the microstructural properties of WM tissue heterogeneity [12, 13]. However, the changes in microstructural properties of the tissue derived from the multishell DTI might be caused by variety of factors, such as axonal density, axonal ordering, degree of myelination, accumulation of pathological proteins, brain atrophy, or microglial activation, and the method does not inform about distinct pathological underlying mechanisms of the observed DTI changes [14].

## 2. Materials and Methods

The cohort consisted of healthy senior volunteers and seniors with mild cognitive impairment (MCI), all potentially capable of participating in the intensive dance intervention. Healthy seniors were recruited using the public media such as local newspapers and radio and TV news. MCI participants were recruited from patients longitudinally followed at the First Department of Neurology, Faculty of Medicine, Masaryk University, Brno, Czech Republic. In brief, we included subjects aged over 60 years, nonsmokers with no alcohol and/or drug abuse, and patients without serious brain injury, dementia, or major depressive disorder. For detailed information about the enrolment and randomization process, see Kropacova et al. [10]. Briefly, 120 participants were randomized to a dance intervention (DI) group and a life as usual (LAU, control) group, 60 participants in each group, using the opaque envelope method.

All subjects underwent the neuropsychological, physical fitness, and MRI examination at the baseline and after 6 months. Informed consent, in accordance with the ethics committee of Masaryk University, was obtained from each subject. The study was approved by the local ethics committee.

### 2.1. Dance Intervention

The dance intervention was organized by specialists from the Faculty of Sports Studies, Masaryk University, Brno, Czech Republic. The intervention took six months and included three training units (each 60 minutes) per week. The whole study lasted for three years with the DI taking place between November and April each year in small groups of up to 20 subjects. The DI program was performed at a medium physical load intensity, and subjects were supervised by an experienced tutor. The load intensity was monitored by the Borg Rating of Perceived Exertion (RPE) scale during each supervised session. The RPE is a user-friendly numerical scale that evaluates an individual's self-reported level of effort, physical exertion, and fatigue during exercise using a 15-point scale ranging from 6 (no exertion) to 20 (maximum exertion) [15]. The physical load was adjusted to the current health condition and physical fitness levels of the individual seniors, and it was kept between 11 and14 points on the RPE. The DI units included folk, country, African, Greek, and tango dancing. The choreographies were divided into smaller blocks that were gradually taught in individual lessons and modified and developed over time into the final choreography. Only subjects who completed at least 60% of the DI program were included in the final cohort [10]. The real average completion of the DI program is 78.1%.

### 2.2. Physical Fitness Examination

The effect of the DI was evaluated by two tests from the functional fitness assessment [16]. The 8-Foot Up-and-Go Test evaluates the agility and dynamic balance. It measures time (in seconds) required to get up from a seated position, walk 8-foot distance, return to the chair, and sit down. The lower values indicate better performance. The 30-Second Chair Stand Test evaluates lower body strength and physical endurance by measuring the number of repetitions of full stands from a chair in 30 seconds. The higher values indicate better performance.

### 2.3. Neuropsychological Examination

Global cognition, five cognitive domains, and activities of daily living were evaluated by complex neuropsychological testing [10]. The examination included the MoCA score [17] and individual tests from the memory domain (Taylor Figure Test [18], Wechsler Memory Scale III: Logical Memory [19]), attention domain (Wechsler Adult Intelligence Scale III: Digit Span, symbol search [20]), executive domain (Five-Point Test [11], Tower of Hanoi [21]), visuospatial domain (Taylor Figure Test [18], Judgement of Line Orientation [22]), language domain (Mississippi Aphasia Screening Test [23]), and activities of daily living (Bristol Activities of Daily Living Scale [24]). The cognitive domain *Z*-scores were calculated as the average *Z*-scores of the tests included in the particular domain [25].

Participants were classified as having MCI if they scored below -1.5 SD in at least two tests in one or more cognitive domains [25]. More specifically, we used the following criteria: MoCA ≥ 26 points and the score below 1.5 SD in two tests in at least one cognitive domain, MoCA < 26 points and the score below 1.5 SD in any two tests, and objective memory deficit on the MoCA and the score below 1.5 SD in at least one test from the memory domain.

### 2.4. DTI-MRI Examination

All subjects were scanned using the 3 T Siemens Prisma MR scanner (Siemens Corp., Erlangen, Germany) in CEITEC Masaryk University, Brno, Czech Republic, employing the following sequences: magnetization-prepared rapid gradient-echo (MPRAGE) high-resolution T1-weighted images (240 sagittal slices, slice thickness = 1 mm, TR = 2300 ms, TE = 2.34 ms, FA = 8°, FOV = 224 mm, and matrix size 224 × 224) and diffusion-weighted images (114 sagittal slices, slice thickness = 2 mm, TR = 9300 ms, TE = 97 ms, and FOV = 228 mm) and thirty noncollinear diffusion directions with *b*-values 500, 1000, and 2000 s/mm^2^, ten T2-weighted acquisitions with *b*-value 0 s/mm^2^, and three acquisitions with *b*-value 0 s/mm^2^ with opposite polarity of phase encoding. FA and mean diffusivity (MD) were the parameters of interest.

### 2.5. Processing of the MRI Data

The structural connectivity of the WM was evaluated using the FSL software [26] and TBSS method [27]. Each subject's raw data was first corrected for susceptibility-induced distortions, eddy current distortions, and movements using the topup [28] and eddy [29] tools. Nonbrain voxels were excluded using the Brain Extraction Toolbox (BET) [30], and the brain extracted masks were checked one by one. Diffusion tensor at each voxel was modeled by DTIFIT function. Maps of FA and MD were calculated. The bedpostx tool was used for modeling with recommended settings [12, 31].

After preprocessing and quality control, the data underwent TBSS [27]. FA images of all subjects were nonlinearly registered to FMRIB58_FA_1mm target image and then affine transformed to the 1 × 1 × 1 mm MNI152 standard space. The mean FA map was calculated, and the skeleton representing the centres of all tracts was created at the threshold 0.2. All individual FA maps were projected onto the skeleton. MD maps were processed using the information from the FA procedure. Individual maps were nonlinearly registered to the common space and projected onto the original mean FA skeleton. Mean values of DTI parameters were extracted from the final WM skeleton.

Paired differences within subjects were calculated, and a two-sample *t*-test design was set in a general linear model (GLM), as suggested by the FSL GLM User Guide. A randomization tool [32] with 5000 permutations was used to calculate the differences between the DI and control groups, controlled for the effect of gender, age, years of education, and the baseline MoCA score.

In addition to the whole-brain WM skeleton exploratory analysis, mean FA and MD were also computed for the corticospinal tract (CST) and the superior longitudinal fascicle (SLF) which are known to be related to motor planning and execution as well as spatial attention and speech comprehension [8, 9, 33, 34]. We used a bidirectional iterative parcellation (BIP) [35] which applies the FSL option of “probabilistic tracking with classification targets” in a bidirectional and iterative manner [35]. The method requires specific gray matter endpoint definition. Initial seed regions and inverse masks were downloaded from BIP's creator Bitbucket depository (https://bitbucket.org/dpat/). Gray matter endpoints for CST were defined as motor-sensory and brainstem. The motor-sensory endpoint mask was created using MARINA [36] by merging masks of the precentral gyrus, postcentral gyrus, and supplementary motor area. The brainstem endpoint originated in the Harvard-Oxford subcortical structural atlas and was truncated at *z* = −20 mm. The gray matter endpoints for SLF were derived from the AAL atlas [37], with the first endpoint being the angular gyrus and the second endpoint being the frontal middle gyrus. We chose specifically the angular gyrus since it is a crossmodal hub where converging multisensory information is combined and integrated to comprehend commands, manipulate mental representations, solve problems, and reorient attention to relevant information [38]. For the probabilistic tractography, the GPU version of Probtrackx was used [39]. The final tracts were thresholded at 5% probability, binarized, and masked with whole-brain WM segmentation. In order to be comparable with the methods and results of a study by Burzynska et al. [9], we additionally segmented the fornix using T1-weighted anatomical images and the FreeSurfer 6.0 (http://surfer.nmr.mgh.harvard.edu) [40] and the mri_cc function.

**Figure 1:**
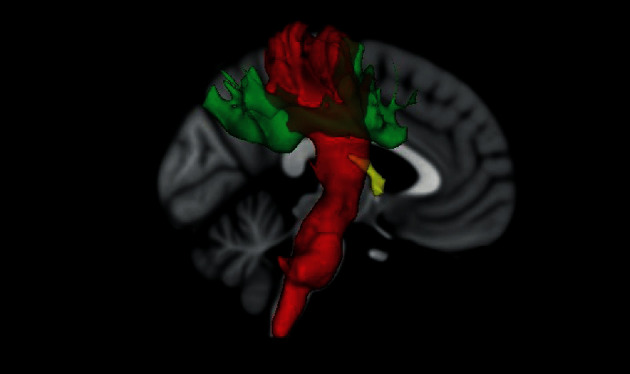
Visualisation of tracts of interest: red—corticospinal tract, green—superior longitudinal fasciculus, and yellow—fornix.

The longitudinal pipeline was used for preprocessing [41]. All segmentations were visually inspected, and 9 subjects (3 DI and 6 LAU) were excluded due to segmentation imprecisions. Binary masks of the fornix were created for each subject and registered to native space DWI b0 images using SPM (https://www.fil.ion.ucl.ac.uk/spm/software/spm12/). For segmentation of all tracts, see Figure 1.

### 2.6. Evaluation of Changes in Cognitive/Physical Fitness Measures of Interest and Changes in DTI Parameters

Equivalency in between-group baseline data was checked by chi-square tests and the Mann-Whitney test.

Mixed ANOVA and the following post-hoc tests were applied to examine DI-induced behavioral, cognitive, and DTI changes, both in the whole brain and in tracts of interest.

Paired *t*-tests were additionally used to test the DI-induced changes in the DI group only.

Partial correlations (Spearman correlation coefficient; MATLAB 2018) were calculated between changes in clinical measures of interest (i.e., those that revealed significant time∗group effects) and changes in FA and MD parameters in the whole-brain WM and above-mentioned tracts of interest in the DI group.

## 3. Results

Altogether, 99 (49 in the DI group and 50 in the LAU group) completed successfully the DI/LAU period. The most common reason to withdraw from the study during the DI period was an unexpected health problem of the participant or his/her partner and problems with keeping up with the time schedule of the intervention. The final research sample with good-quality clinical, cognitive, and diffusion MRI data both at the baseline and at the follow-up examination after the DI/LAU consisted of 76 participants (51 HC and 25 MCI subjects). At the baseline, groups in the final cohort were not significantly different in age, years of education, and relative proportion of MCI participants. We found between-group differences at the baseline visit in gender and in the global cognitive MoCA score. Gender differences were due to the dropout rate, which was clearly gender-related in the DI group: fewer men than women completed the DI program. Therefore, we controlled all results for the effect of gender, age, years of education, and the baseline MoCA score. All cognitive, physical fitness, and DTI data at the baseline visit and at the follow-up visit after six months in the DI and control groups are depicted in Table 1. All cognitive, physical fitness, and DTI data at the baseline visit and at the follow-up visit after six months in both groups, divided into HC and MCI subgroups, are depicted in Supplementary Material Tables S1 and S2.

### 3.1. Behavioral Results

**Figure 2:**
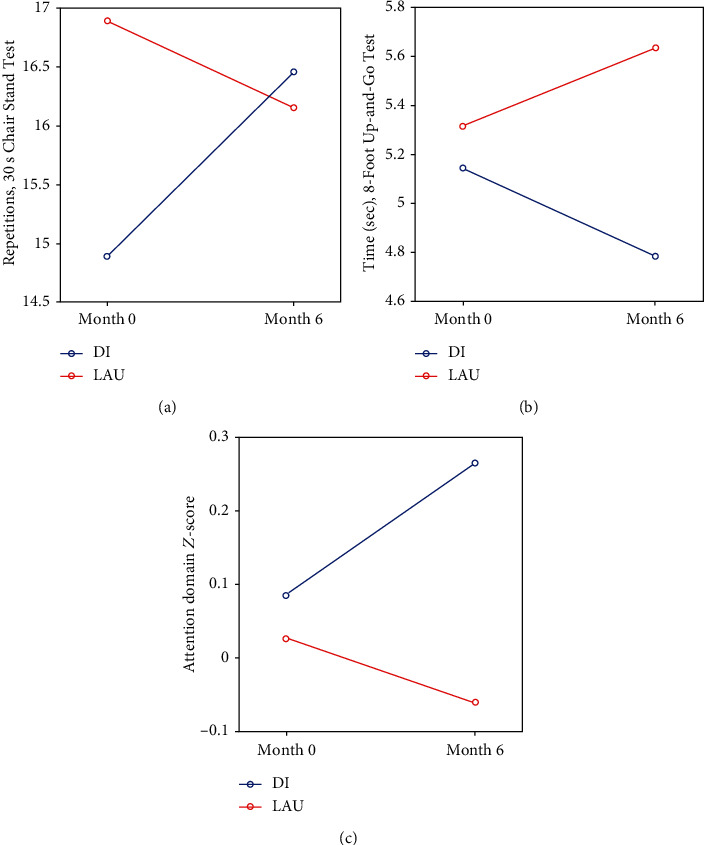
Mixed ANOVA results: significant time∗group changes. DI: dance intervention; LAU: life as usual. (a) Number of repetitions, 30-Second Chair Stand Test (increase means improvement); (b) time, 8-Foot Up-and-Go Test (decrease means improvement); and (c) attention domain *Z*-score.

Mixed ANOVA revealed a significant time∗group effect (see Figure 2), in the 8-Foot Up-and-Go Test (*p* = 0.006) and in the 30-Second Chair Stand Test (*p* = 0.021). There was an effect in the attention domain (*p* = 0.015), but it did not survive the FDR correction for five cognitive domains. Complete results can be found in Supplementary Material Table S3.

Paired *t*-tests in the DI group revealed significant DI-induced changes (improvement) in the 30-Second Chair Stand Test (*p* = 0.002) and in the 8-Foot Up-and-Go Test (*p* = 0.014). The DI also led to improved attention (*p* = 0.033) and executive domain *Z*-score (*p* = 0.007) in the DI group, but the results did not survive the FDR correction for five cognitive domains. For complete results, see Supplementary Material Table S4.

### 3.2. DTI Results in the Whole-Brain WM and in the WM Tracts of Interest and Relation to Physical Fitness

TBSS showed no significant differences between the DI and control groups. As for the DTI measures in the subanalyses of the tracts of interest, no significant changes in FA and MD were observed between the two groups (for details, see Supplementary Material Table S3).

**Figure 3:**
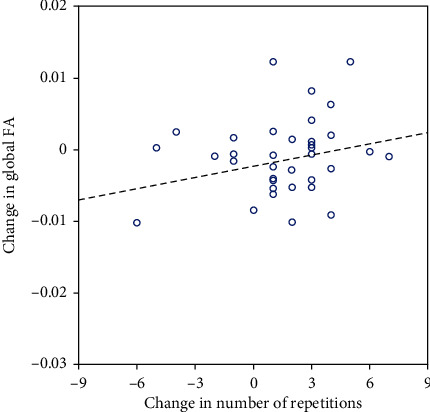
Relationship between change in FA and change in performance in the 30-Second Chair Stand Test.

Partial correlation analysis showed a significant relationship between the performance in the 30-Second Chair Stand Test and global WM FA (*p* = 0.016, *R* = 0.41) in the DI group (see Figure 3).

Additional analyses in the tracts of interest in the DI group showed positive medium strength correlations between FA in the left SLF and performance in the 30-Second Chair Stand Test (*p* = 0.006, *R* = 0.47) and between MD and FA in the right CST and time to perform the 8-Foot Up-and-Go Test (*p* = 0.006, *R* = 0.46 and *p* = 0.023, *R* = −0.39, respectively). For complete results, see Supplementary Material Table S5.

## 4. Discussion

Our study focused on WM microstructure correlates of behavioral and cognitive effects of intensive six-month dance exercise training in mixed nondemented seniors including cognitively intact individual as well as a small proportion of MCI subjects.

We observed DI-induced effects in the physical fitness measures, namely, the 8-Foot Up-and-Go Test and 30-Second Chair Stand Test, in the DI group as compared to the control group. A better performance in these tests means improvements in the dynamic balance, agility, lower body strength, and physical endurance. These parameters are key factors in preserving mobility and independence in older age [16]. A positive effect of DI on physical fitness was consistently reported by others and was summarized in a review by Hwang and Braun [1]. On the other hand, the effects of DI on cognition are more heterogeneous and have remained inconsistent. Some authors reported a positive DI-induced effect on attention [5, 6, 42, 43] and memory [42, 43] functions, but positive changes were reported also in groups who underwent conventional fitness training [42, 43]. In this DTI substudy, we observed a DI-induced effect solely on the attention domain, but this result did not survive the FDR correction and therefore should be interpreted with caution. We did not observe any DI-induced effects between both groups in the microstructure in the WM skeleton or in tracts of interest.

Our major study aim was to identify whether WM microstructural changes, as assessed by FA and MD measures derived from a multishell diffusion protocol, may underlie dance-induced behavioral improvements. FA is a directionally dependent sensitive marker of microstructural changes (e.g., myelination, fiber orientation, and axonal diameter) [14]. Increase in FA can be explained by, e.g., higher packing density or increased directional organization of axons and/or stabilization or increase in myelin integrity [14, 44]. MD is a directionally independent measure that describes overall diffusion within the voxel [45]; increased MD is characteristic for regions where neural microstructures (e.g., axonal cell membranes, myelin sheaths, and neurofilaments) are displaced by intra- and extracellular water [46]. Studies showed that the increase in MD and decrease in FA are the common process in healthy aging [47]. The WM integrity also reflects age-related variability in cognitive outcomes in healthy aged individuals [48] such that increased FA/decreased MD relates to increased cognitive performance [46].

A positive relationship between improved physical fitness and increased WM integrity in this study was detected in the whole brain as well as in the WM tracts of interest related to motor learning and movement execution, as well as to spatial attention, manipulation of mental representations, and speech comprehension. The CST projects from the motor cortices to the spinal cord and plays a key role in the control of voluntary movement [34]. The SLF is considered to be the major cortical association fiber pathway. As for the particular results of correlation with increased physical endurance, the SLF plays a role in regulating motor learning and higher aspects of motor behavior [33, 34]. Based on the results of Burzynska et al. [9], we also focused on the fornix, the major efferent tract of the hippocampus with a key function of memory formation and consolidation [49], although engagement in motor functions in normal aging has also been described [50]. Unlike Burzynska et al. [9], we did not observe any relationship between changes in DTI measures within this tract and cognitive functions or physical fitness.

Our results were similar to those of Voss et al. [8] who did not find significant DTI changes on a group level even after a whole year of the aerobic intervention (walking performed three times per week). The authors observed that increased FA in the WM of prefrontal, parietal, and temporal areas (no tracts were specified in this work) was related to changes in cardiorespiratory fitness measures in the walking intervention group. However, significant associations were not supported by the TBSS analysis. No specific tracts were analysed.

Some novel methodological aspects of the current study should be highlighted: we used the multishell diffusion MRI protocol which is thought to surpass conventional single-shell DTI in terms of its ability to accurately evaluate microstructural properties with varied restrictions to diffusion [13]. In addition to TBSS, tracts of interest were delineated including the probabilistic tracking method applicable for evaluating long association/projection pathways [35]. However, despite using these methods, we were not able to identify changes in WM integrity due to the DI.

It would have been interesting to examine the HC and MCI subjects separately. However, this was not possible due to a small proportion of MCI subjects in DI and LAU cohorts.

## 5. Study Limitations

The distribution of demographic and cognitive characteristics and the number of MCI subjects in both groups were comparable in the whole cohort of 99 participants [10]. However, some subjects had to be discarded because of incomplete or low-quality diffusion MRI data. This led to rather disproportional distribution of MCI subjects in the groups of this DTI substudy although the number of MCI subjects in the DI and LAU groups was not significantly different.

The number of MCI patients in DI and LAU groups was too low to perform separate analysis for MCI.

The MoCA score was lower in the LAU group as compared to the DI group. Despite the fact that we controlled for the effect of the baseline MoCA score in our further analyses, we cannot fully exclude a possible effect of unequally distributed MCI participants in both groups. Another limitation of the study is a rather low number of applied diffusion directions for probabilistic tractography analysis.

## 6. Conclusion

In conclusion, we showed that 6 months of intensive DI can increase physical fitness measures evaluating lower body muscle endurance, agility, and balance in aged nondemented individuals and is associated with the enhancement in structural integrity, particularly in specific tracts that are engaged in motor behavior, regulation of motor learning, and coordination and control of voluntary movement. Future studies should focus on possible differences in the behavioral effects of DI and related DTI changes separately in the groups of heathy seniors and MCI patients.

## Figures and Tables

**Table 1:** (a) Descriptive characteristics. (b) Physical fitness, cognitive, and DTI data at the baseline. (c) Physical fitness, cognitive, and DTI data after 6 months. Mean ± standard deviation. ^∗^Significant difference.

	DI group	Control group	*p* value
(a) Descriptive characteristics			
*N*	37	39	—
Gender	32 F/5 M	26 F/13 M	0.042^∗^
Age	69.3 ± 5.3	68.9 ± 6.3	0.607
Years of education	15.0 ± 2.2	15.0 ± 3.0	0.084
HC/MCI	28/9	23/16	0.121
(b) Baseline—physical fitness, cognitive, and DTI data			
8-Foot Up-and-Go Test (seconds)	5.1 ± 1.5	5.3 ± 1.4	0.890
30-Second Chair Stand Test (number of repetitions)	14.9 ± 4.0	16.8 ± 4.8	0.191
MoCA	27.4 ± 2.7	25.8 ± 2.7	0.004^∗^
Memory (*Z*-score)	1.11 ± 1.01	0.93 ± 0.89	0.374
Attention (*Z*-score)	0.08 ± 0.58	0.02 ± 0.71	0.559
Executive (*Z*-score)	−0.35 ± 0.63	−0.30 ± 0.74	0.831
Visuospatial (*Z*-score)	0.29 ± 0.55	0.38 ± 0.56	0.417
Language (*Z*-score)	0.39 ± 0.46	0.40 ± 0.42	0.872
Global WM FA	0.43 ± 0.02	0.42 ± 0.02	0.979
Global WM MD (mm^2^s^−1^)	0.0007 ± 0.00002	0.0007 ± 0.00005	0.230
(c) After 6 months—physical fitness, cognitive, and DTI data			
8-Foot Up-and-Go Test (seconds)	4.8 ± 1.2	5.6 ± 2.3	0.537
30-Second Chair Stand Test (number of repetitions)	16.4 ± 4.1	16.2 ± 6.3	1.000
MoCA	27.0 ± 2.7	26.6 ± 2.5	0.367
Memory (*Z*-score)	1.30 ± 0.77	1.15 ± 0.92	0.318
Attention (*Z*-score)	0.26 ± 0.71	−0.06 ± 0.75	0.046^∗^
Executive (*Z*-score)	−0.02 ± 0.82	−0.22 ± 0.65	0.278
Visuospatial (*Z*-score)	0.30 ± 0.59	0.48 ± 0.55	0.166
Language (*Z*-score)	0.46 ± 0.43	0.42 ± 0.51	0.826
Global WM FA	0.43 ± 0.02	0.43 ± 0.02	0.751
Global WM MD (mm^2^s^−1^)	0.0007 ± 0.00002	0.0007 ± 0.00005	0.238

## Data Availability

Anonymised imaging data of this study will be available on reasonable request from any qualified researcher, following the EU General Data Protection Regulation.

## Supplementary Materials

Supplementary Materials Table S1: DI group and HC and MCI subgroups; mean ± standard deviation. Table S2: LAU group and HC and MCI subgroups; mean ± standard deviation. Table S3: mixed ANOVA—behavioral, cognitive (FDR-corrected), and DTI (FDR-corrected) results. ^∗^Significant. WM: white matter; CST: corticospinal tract; SLF: superior longitudinal fasciculus; FA: fractional anisotropy; MD: mean diffusivity. Table S4: paired *t*-tests: DI-induced changes in the DI group—behavioral, cognitive (FDR-corrected), and DTI (FDR-corrected) results; mean ± standard deviation. ^∗^Significant. WM: white matter; CST: corticospinal tract; SLF: superior longitudinal fasciculus; FA: fractional anisotropy; MD: mean diffusivity. MD unit (10^−3^ mm^2^ s^−1^). Table S5: partial correlations (Spearman correlation coefficient; MATLAB 2018) between changes in clinical measures of interest (i.e., those that revealed significant time∗group effects) and changes in FA and MD parameters in the WM in the DI group; *p* value/correlation coefficient. ^∗^Significant. WM: white matter; CST: corticospinal tract; SLF: superior longitudinal fasciculus; FA: fractional anisotropy; MD: mean diffusivity.
