# Supplementary material for: Multishell Diffusion MRI Reflects Improved Physical Fitness Induced by Dance Intervention
Source: Neural Plast. 2020 Nov 5;2020:8836925. doi: 10.1155/2020/8836925 (PMC7661125; doi:10.1155/2020/8836925)
Supplement: Supplementary Materials — Table S1: DI group and HC and MCI subgroups; mean ± standard deviation. Table S2: LAU group and HC and MCI subgroups; mean ± standard deviation. Table S3: mixed ANOVA—behavioral, cognitive (FDR-corrected), and DTI (FDR-corrected) results. ∗Significant. WM: white matter; CST: corticospinal tract; SLF: superior longitudinal fasciculus; FA: fractional anisotropy; MD: mean diffusivity. Table S4: paired t-tests: DI-induced changes in the DI group—behavioral, cognitive (FDR-corrected), and DTI (FDR-corrected) results; mean ± standard deviation. ∗Significant. WM: white matter; CST: corticospinal tract; SLF: superior longitudinal fasciculus; FA: fractional anisotropy; MD: mean diffusivity. MD unit (10−3 mm2 s−1). Table S5: partial correlations (Spearman correlation coefficient; MATLAB 2018) between changes in clinical measures of interest (i.e., those that revealed significant time∗group effects) and changes in FA and MD parameters in the WM in the DI group; p value/correlation coefficient. ∗Significant. WM: white matter; CST: corticospinal tract; SLF: superior longitudinal fasciculus; FA: fractional anisotropy; MD: mean diffusivity. [file 8836925.f1.zip › 8836925 -Supplementary description.docx]

Supplementary description

Table S1 DI group, HC and MCI subgroups; mean ± standard deviation

Table S2 LAUgroup, HC and MCI subgroups; mean ± standard deviation

Table S3Mixed ANOVA – behavioral, cognitive (FDR corrected) and DTI (FDR corrected) results; * significant; WM – whitematter, CST – corticospinaltract, SLF – superior longitudinalfasciculus, FA – fractionalanisotropy, MD – meandiffusivity

Table S4Paired t-tests – DI inducedchanges in the DI group - behavioral, cognitive (FDR corrected) and DTI (FDR corrected) results; mean ± standard deviation; * significant; WM – whitematter, CST – corticospinaltract, SLF – superior longitudinalfasciculus, FA – fractionalanisotropy, MD – meandiffusivity; MD unit (10^-3^mm^2^s^-1^)

Table S5Partial correlations (Spearman correlation coefficient; MATLAB 2018) between changes in clinical measures of interest (i.e. those that revealed significant time*group effects) and changes in FA and MD parameters in the WM in the DI group; p-value / correlation coefficient; * significant; WM – whitematter, CST – corticospinaltract, SLF – superior longitudinalfasciculus, FA – fractionalanisotropy, MD – meandiffusivity
