# Supplementary material for: Multishell Diffusion MRI Reflects Improved Physical Fitness Induced by Dance Intervention
Source: Neural Plast. 2020 Nov 5;2020:8836925. doi: 10.1155/2020/8836925 (PMC7661125; doi:10.1155/2020/8836925)
Supplement: Supplementary Materials — Table S1: DI group and HC and MCI subgroups; mean ± standard deviation. Table S2: LAU group and HC and MCI subgroups; mean ± standard deviation. Table S3: mixed ANOVA—behavioral, cognitive (FDR-corrected), and DTI (FDR-corrected) results. ∗Significant. WM: white matter; CST: corticospinal tract; SLF: superior longitudinal fasciculus; FA: fractional anisotropy; MD: mean diffusivity. Table S4: paired t-tests: DI-induced changes in the DI group—behavioral, cognitive (FDR-corrected), and DTI (FDR-corrected) results; mean ± standard deviation. ∗Significant. WM: white matter; CST: corticospinal tract; SLF: superior longitudinal fasciculus; FA: fractional anisotropy; MD: mean diffusivity. MD unit (10−3 mm2 s−1). Table S5: partial correlations (Spearman correlation coefficient; MATLAB 2018) between changes in clinical measures of interest (i.e., those that revealed significant time∗group effects) and changes in FA and MD parameters in the WM in the DI group; p value/correlation coefficient. ∗Significant. WM: white matter; CST: corticospinal tract; SLF: superior longitudinal fasciculus; FA: fractional anisotropy; MD: mean diffusivity. [file 8836925.f1.zip › Sejnoha_Minsterova_supplementary.docx]

**Supplementary material**

| **DI group: Descriptive characteristics** | | |
| --- | --- | --- |
|  | **HC** | **MCI** |
| N | 28 | 9 |
| Gender | 24F/4M | 8F/1M |
| Age | 68.9 ± 5.2 | 70.4 ± 6.2 |
| Years of education | 15.0 ± 2.4 | 13.9 ± 1.5 |
| **Baseline – Physical fitness, cognitive and DTI data** | | |
| 8-foot up-and-go test (seconds) | 5.0 ± 1.6 | 5.3 ± 0.1 |
| 30-s chair stand test (number of repetitions) | 15.1 ± 4.3 | 14.3 ± 3.4 |
| MoCA | 28.5 ± 1.4 | 24.0 ± 3.0 |
| Memory (Z-score) | 1.43 ± 0.62 | 0.14 ± 1.37 |
| Attention (Z-score) | 0.26 ± 0.44 | -0.48 ± 0.60 |
| Executive (Z-score) | -0.15 ± 0.51 | -0.96 ± 0.61 |
| Visuospatial (Z-score) | 0.40 ± 0.54 | -0.03 ± 0.48 |
| Language (Z-score) | 0.51 ± 0.39 | 0.04 ± 0.54 |
| Global WM FA | 0.43 ± 0.02 | 0.43 ± 0.02 |
| Global WM MD (mm^2^s^-1^) | 0.0007 ± 0.00002 | 0.0007 ± 0.00003 |
| **After 6 months - Physical fitness, cognitive and DTI data** | | |
| 8-foot up-and-go test (seconds) | 4.7 ± 1.3 | 4.8 ± 0.42 |
| 30-s chair stand test (number of repetitions) | 16.7 ± 4.5 | 15.6 ± 2.2 |
| MoCA | 27.5 ± 2.0 | 25.6 ± 3.9 |
| Memory (Z-score) | 1.45 ± 0.44 | 0.83 ± 1.23 |
| Attention (Z-score) | 0.46 ± 0.61 | -0.34 ± 0.65 |
| Executive (Z-score) | 0.22 ± 0.61 | -0.81 ± 0.96 |
| Visuospatial (Z-score) | 0.42 ± 0.58 | -0.07 ± 0.48 |
| Language (Z-score) | 0.47 ± 0.47 | 0.43 ± 0.36 |
| Global WM FA | 0.43 ± 0.02 | 0.43 ± 0.02 |
| Global WM MD (mm^2^s^-1^) | 0.0007 ± 0.00002 | 0.0007 ± 0.00003 |

Table S1 DI group, HC and MCI subgroups; mean ± standard deviation

| **LAU group: Descriptive characteristics** | | |
| --- | --- | --- |
|  | **HC** | **MCI** |
| N | 23 | 16 |
| Gender | 17F/6M | 9F/7M |
| Age | 68.3 ± 6.5 | 69.7 ± 6.4 |
| Years of education | 15.4 ± 2.8 | 14.3 ± 3.4 |
| **Baseline – Physical fitness, cognitive and DTI data** | | |
| 8-foot up-and-go test (seconds) | 5.1 ± 1.5 | 5.5 ± 1.3 |
| 30-s chair stand test (number of repetitions) | 16.8 ± 4.3 | 17.0 ± 5.8 |
| MoCA | 26.3 ± 2.2 | 25.2 ± 3.4 |
| Memory (Z-score) | 1.31 ± 0.60 | 0.38 ± 0.98 |
| Attention (Z-score) | 0.18 ± 0.76 | -0.19 ± 0.60 |
| Executive (Z-score) | -0.09 ± 0.66 | -0.59 ± 0.78 |
| Visuospatial (Z-score) | 0.58 ± 0.41 | 0.10 ± 0.64 |
| Language (Z-score) | 0.29 ± 0.49 | 0.57 ± 0.26 |
| Global WM FA | 0.43 ± 0.02 | 0.43 ± 0.02 |
| Global WM MD (mm^2^s^-1^) | 0.0007 ± 0.00005 | 0.0007 ± 0.0006 |
| **After 6 months - Physical fitness, cognitive and DTI data** | | |
| 8-foot up-and-go test (seconds) | 5.7 ± 2.8 | 5.5 ± 1.4 |
| 30-s chair stand test (number of repetitions) | 15.8 ± 5.9 | 16.5 ± 6.8 |
| MoCA | 27.5 ± 2.2 | 25.3 ± 2.4 |
| Memory (Z-score) | 1.55 ± 0.70 | 0.57 ± 0.92 |
| Attention (Z-score) | 0.2 ± 0.77 | -0.42 ± 0.56 |
| Executive (Z-score) | -0.03 ± 0.58 | -0.49 ± 0.67 |
| Visuospatial (Z-score) | 0.63 ± 0.43 | 0.27 ± 0.65 |
| Language (Z-score) | 0.41 ± 0.55 | 0.43 ± 0.47 |
| Global WM FA | 0.42 ± 0.02 | 0.43 ± 0.3 |
| Global WM MD (mm^2^s^-1^) | 0.0007 ± 0.00005 | 0.0007 ± 0.00006 |

Table S2 LAU group, HC and MCI subgroups; mean ± standard deviation

| **Test/domain/tract** | **time*group effect p-value** |
| --- | --- |
| The 8-Foot Up-and-Go Test | 0.006* |
| 30-s Chair Stand Test | 0.021* |
| Memory domain | 0.880 |
| Attention domain | 0.015 |
| Executive domain | 0.111 |
| Visuospatial domain | 0.455 |
| Language domain | 0.600 |
| Global WM FA | 0.925 |
| Global WM MD | 1.000 |
| CST FA R | 0.949 |
| CST MD R | 1.000 |
| CST FA L | 0.260 |
| CST MD L | 1.000 |
| SLF FA R | 0.203 |
| SLF MD R | 0.550 |
| SLF FA L | 0.281 |
| SLF MD L | 0.575 |
| Fornix FA | 0.052 |
| Fornix MD | 0.376 |

Table S3 Mixed ANOVA – behavioral, cognitive (FDR corrected) and DTI (FDR corrected) results; * significant; WM – white matter, CST – corticospinal tract, SLF – superior longitudinal fasciculus, FA – fractional anisotropy, MD – mean diffusivity

| **Test/domain** | **Baseline** | **After DI** | **p-value** |
| --- | --- | --- | --- |
| The 8-Foot Up-and-Go Test (second) | 5.1 ± 1.5 | 4.8 ± 1.2 | 0.014* |
| 30-s Chair Stand Test (number of repetitions) | 14.9 ± 4.0 | 16.4 ± 4.1 | 0.002* |
| Memory domain (Z-score) | 1.11 ± 1.01 | 1.30 ± 0.77 | 0.072 |
| Attention domain (Z-score) | 0.08 ± 0.58 | 0.26 ± 0.71 | 0.033 |
| Executive domain (Z-score) | -0.35 ± 0.63 | -0.02 ± 0.82 | 0.007 |
| Visuospatial domain (Z-score) | 0.29 ± 0.55 | 0.30 ± 0.59 | 0.922 |
| Language domain (Z-score) | 0.39 ± 0.46 | 0.46 ± 0.43 | 0.444 |
| Global WM FA | 0.43 ± 0.02 | 0.43 ± 0.02 | 0.069 |
| Global WM MD | 0.72 ± 0.03 | 0.72 ± 0.03 | 0.381 |
| CST FA R | 0.51 ± 0.02 | 0.51 ± 0.02 | 0.547 |
| CST MD R | 0.61 ± 0.02 | 0.60 ± 0.02 | 0.067 |
| CST FA L | 0.51 ± 0.02 | 0.51 ± 0.02 | 0.835 |
| CST MD L | 0.60 ± 0.02 | 0. 60 ± 0.02 | 0.374 |
| SLF FA R | 0.47 ± 0.03 | 0.47 ± 0.03 | 0.388 |
| SLF MD R | 0.65 ± 0.03 | 0.65 ± 0.03 | 0.916 |
| SLF FA L | 0.48 ±0.03 | 0.48 ± 0.03 | 0.504 |
| SLF MD L | 0.65 ± 0.03 | 0.65 ± 0.03 | 0.708 |
| Fornix FA | 0.42 ± 0.05 | 0.41 ± 0.06 | 0.076 |
| Fornix MD | 0.88 ± 0.06 | 0.88 ± 0.05 | 0.769 |

Table S4 Paired t-tests – DI induced changes in the DI group - behavioral, cognitive (FDR corrected) and DTI (FDR corrected) results; mean ± standard deviation; * significant; WM – white matter, CST – corticospinal tract, SLF – superior longitudinal fasciculus, FA – fractional anisotropy, MD – mean diffusivity; MD unit (10^-3^mm^2^s^-1^)

|  | **The 8-Foot Up-and-Go Test** | **30-s Chair Stand Test** |
| --- | --- | --- |
| Global WM FA | 0.48 / -0.12 | 0.02* / 0.41 |
| Global WM MD | 0.97 / -0.01 | 0.39 / 0.15 |
| CST FA R | 0.02* / -0.39 | 0.27 / 0.20 |
| CST MD R | >0.01* / 0.46 | 0.17 / 0.24 |
| CST FA L | 0.47 / -0.12 | 0.19 / 0.23 |
| CST MD L | 0.21 / 0.22 | 0.79 / 0.05 |
| SLF FA R | 0.36 / -0.16 | 0.29 / 0.19 |
| SLF MD R | 0.53 / 0.11 | 0.46 / 0.13 |
| SLF FA L | 0.88 / -0.02 | >0.01* / 0.47 |
| SLF MD L | 0.30 / -0.19 | 0.26 / 0.20 |
| Fornix FA | 0.23 / 0.22 | 0.62/ 0.09 |
| Fornix MD | 0.54 / 0.11 | 0.78 / -0.05 |

Table S5 Partial correlations (Spearman correlation coefficient; MATLAB 2018) between changes in clinical measures of interest (i.e. those that revealed significant time*group effects) and changes in FA and MD parameters in the WM in the DI group; p-value / correlation coefficient; * significant; WM – white matter, CST – corticospinal tract, SLF – superior longitudinal fasciculus, FA – fractional anisotropy, MD – mean diffusivity
